# Supplementary material for: Transcriptomics Reveal Molecular Differences in Equine Oocytes Vitrified before and after In Vitro Maturation
Source: Int J Mol Sci. 2023 Apr 7;24(8):6915. doi: 10.3390/ijms24086915 (PMC10138936; doi:10.3390/ijms24086915)
Supplement: Supplementary file 1 [file ijms-24-06915-s001.zip › Supplementary Table S1.pdf]

Supplementary Table S1. Survival, maturation, and efficiency rate in fresh and vitrified oocytes.

Group	Survival rate	Maturation Rate	Efficiency
Fresh	111/111 (100%)	111/190 (58.3 ± 3.1%)[a*]	111/190 (58.3 ± 3.6%)[a]
VMAT	112/135 (87.8 ± 2.9%)	135/232 (58.3 ± 3.1%)[a*]	112/232 (48.3 ± 3.3%)[ab]
VIM	204/210 (97.7 ± 1.1%)	84/204 (40.5 ± 4.4%;)[b]	84/210 (40.0 ± 3.4%)[b]

The survival rate is defined as the number of oocytes with an intact oolema after warming/ number of vitrified oocytes. The maturation rate is defined as the number of oocytes with an extruded polar body/ number of oocytes exposed to in vitro maturation. The efficiency was calculated as the number of oocytes stored for transcriptomics/initial number of oocytes. Groups with different superscripts differ significantly ($p < 0.05$). * The maturation rate in fresh and VMAT is equal, since oocytes for these groups were divided after in vitro maturation, and both underwent in vitro maturation together (n = 422). Therefore, the initial number (fresh n = 190 and VMAT n = 232) was deducted from the maturation rate (58.3%) of the oocytes assigned to group 1: fresh non-vitrified oocytes as a control (FR); n = 111) or group 2: oocytes vitrified after in vitro maturation (VMAT); n = 135) after in vitro maturation.”. Results are presented as n (least square means ± standard error). VIM: oocytes vitrified before in vitro maturation.
